# Total Reconstruction of the Auricle: Our Experiences on Indications and Recent Techniques

**DOI:** 10.1155/2014/373286

**Published:** 2014-04-14

**Authors:** K. Storck, R. Staudenmaier, M. Buchberger, T. Strenger, K. Kreutzer, A. von Bomhard, T. Stark

**Affiliations:** ^1^Department of Otorhinolaryngology, Head and Neck Surgery, Klinikum Rechts der Isar, Technische Universität München, Ismaninger Strasse 22, 81675 München, Germany; ^2^Department of Maxillofacial Surgery, Technische Universität München, Ismaninger Strasse 22, 81675 München, Germany

## Abstract

*Introduction*. Auricular reconstruction is a great challenge in facial plastic surgery. With the advances in surgical techniques and biotechnology, different options are available for consideration. The aim of this paper is to review the knowledge about the various techniques for total auricular reconstruction based on the literature and our experience. *Methods*. Approximately 179 articles published from 1980 to 2013 were identified, and 59 articles were included. We have focused on the current status of total auricular reconstruction based on our personal experience and on papers of particular interest, published within the period of review. We have also included a prospective view on the tissue engineering of cartilage. *Results*. Most surgeons still practice total auricular reconstruction by employing techniques developed by Brent, Nagata, and Firmin with autologous rib cartilage. Within the last years, alloplastic frameworks for reconstruction have become well established. Choosing the reconstruction techniques depends mainly on the surgeon's preference and experience. Prosthetic reconstruction is still reserved for special conditions, even though the material is constantly improving. Tissue engineering has a growing potential for clinical applicability. *Conclusion*. Auricular reconstruction still receives attention of plastic/maxillofacial surgeons and otolaryngologists. Even though clinical applicability lags behind initial expectations, the development of tissue-engineered constructs continues its potential development.

## 1. Introduction

The field of auricular reconstruction is still a huge challenge for facial plastic surgeons and requires a broad view of various techniques in order to find the best treatment for each patient. Auricular defects and deformities include not only acquired defects attributable to trauma, burns, tumours, piercing defects, scars, and inflammation/allergies, but also congenital auricular malformations ranging from Grade I malformation (e.g., prominent ears) to Grade III malformations including severe microtia changes. Often, they are accompanied by aural atresia, malformations of the middle ear, and sometimes even facial anomalies with the facial nerve being affected on the ipsilateral side.

Depending on the defect and the surrounding circumstances, the restoration of a fully satisfactory, complete auricle is always the main goal of the patient and the plastic surgeon. With progress both in surgical techniques and in biotechnology, the range of possibilities of using costal cartilage grafts, alloplastic materials, or a prosthesis is becoming wider. Moreover, advances in the tissue engineering of cultured cartilage will be of clinical interest in the future. The first known described operation of microtia Grade III was carried out in 1870 by von Syzmanowski [1]. However, techniques have greatly changed since then. The major factors that influence the treatment options include each patient's pathology, local tissue, and skin conditions and the preferences of the facial plastic surgeon and the patient.

Since the main aim of the plastic surgeon should always be to the benefit of the patient with an associated improved quality of life, this paper is concerned with the current knowledge of the various management options of congenital and acquired (sub-)total defects of the auricle based on the recent literature. It also includes the promising field of the tissue engineering of cultured cartilage as reported in recent publications. The information provided in this article should help the surgeon with the decision-making process regarding the best use of the available materials for total reconstruction.

## 2. Material and Methods

The MEDLINE database was used to search for all English and non-English studies involving total auricular reconstruction and the outcome of various techniques. We employed the search term (microtia or auricular amputation or external ear injury) and reconstruction (autologous or alloplastic or Medpor or prosthetic). One hundred and seventy-nine articles were identified and the abstracts were evaluated. Focusing on total reconstruction with clinical applicability, we excluded animal studies, all review articles, and studies published before 1980. Studies were also excluded if they were unable to be translated, involved partial reconstructions (not the main focus of this paper), or were concerned with other or multiple facial reconstructions since the decision-making process could be influenced by the other defects.

**Figure 1:**
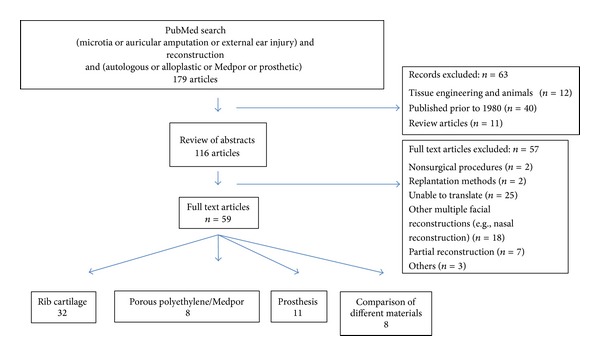
MEDLINE database search resulting in 59 publications concerned with total auricular reconstruction.

Finally, 59 articles focusing on reconstruction via rib cartilage (*n* = 32), porous polyethylene (*n* = 8), prosthesis (*n* = 11), or a comparison of various materials (*n* = 8) were found (Figure 1). Of all the articles, we included papers of particular interest mainly based on our own experience. Other sources of information were mainly conference proceedings and personal communications. Our main interest was the current status of total auricular reconstruction via diverse alternatives such as autologous, alloplastic, or prosthetic material. Their pros and cons were recorded, in addition to current experimental knowledge of the tissue engineering of cartilage, with the main goal of coming closer to clinical applicability (this topic was not included in the main PubMed research, since clinical applicability is generally still lacking). For the sake of completeness, we also include a short overview of partial defects.

## 3. Results

### 3.1. Anatomy

#### 3.1.1. Normal Auricle

**Figure 2:**
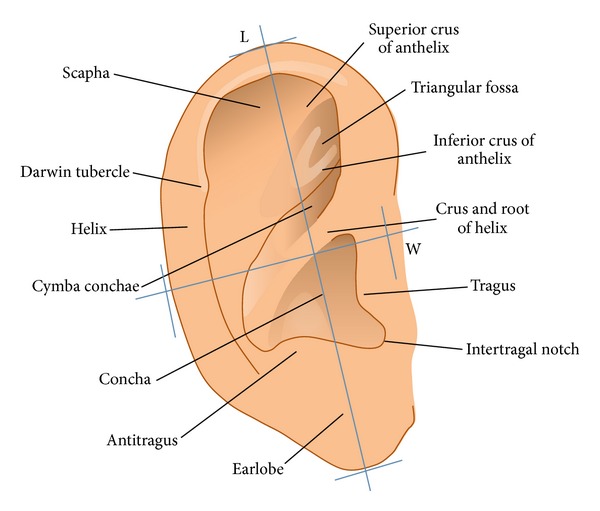
Anatomy and landmarks of the auricle.

The size of the normal auricle depends on various factors such as age, gender, and body height (Figure 2). In adults, the length (*L*) is about 58–66 mm (women 58–63 mm, men 62–66 mm) and the width (*W*) approximately 32–34 mm. By the age of 8 years, the auricle has reached its full size, an important consideration in the reconstruction of the ear in children.

## 4. Auricular Deformities

In a study by Guo et al. [2], the complete reconstruction of the auricle via a prosthesis in 46 patients was undertaken because of congenital deformities (65.2%), tumour resection (26.1%), trauma (4.3%), burns (2.2%), and infection (2.2%).

### 4.1. Acquired Deformities

#### 4.1.1. Tumours

Malignant lesions of the external ear account for approximately 6% of all head and neck skin cancers and can be derived from all kinds of tissues in this region, such as the skin, vessels, nerves, bone, and cartilage. The most common malignancies are spinal cell carcinomas and basal cell carcinomas. In these cases, older men with a history of excessive sun exposure are usually affected. Rarely seen cancers include malignant melanomas, keratoacanthoma, adenocarcinomas, adenoidzystic-carcinomas, Merkel-cell carcinomas, angiosarcoma, Bowen's disease, or generalised neoplastic changes such as Kaposi sarcoma. However, benign tumours such as the relapsing polychondritis, gout tophus, neurofibroma, and keloids might also require surgical treatment. In addition, exposure to exogenic stimulations and immune suppression can trigger malignancies. Depending on the entity and the size of the tumour, the defect can vary enormously and necessitates diverse strategies of reconstruction.

#### 4.1.2. Trauma and (Non)-Inflammatory Processes: [3]

The exposed position of the auricle makes it vulnerable to many kinds of injuries.

Acute trauma of the auricle includes chemical burns. Acid burns result in superficial injuries and alkali burns produce more penetrating injuries. Thermal injuries include scalds from hot liquids and exposure to flames, gas explosions, and steam and burns from accidents. Depending on the degree of the burn, the auricle might be affected to levels ranging from a merely painful erythema to a complete loss of the structure of the auricle. Acute trauma also includes otoseroma and otohematomas.

Auricular injuries include blunt traumata by abrasion, tear, and avulsion of the auricle with decession of the skin and perichondrium. However, bite and piercing injuries and inflammation/allergies can affect the auricle to various degrees. Luo et al. [4] have described a distribution of trauma injuries in 60 patients, with 35/60 burns, 10/60 traffic accidents, 8/60 cuts during fights, and 7/60 human bite injuries.

#### 4.1.3. Classification of Acquired Defects

**Figure 3:**
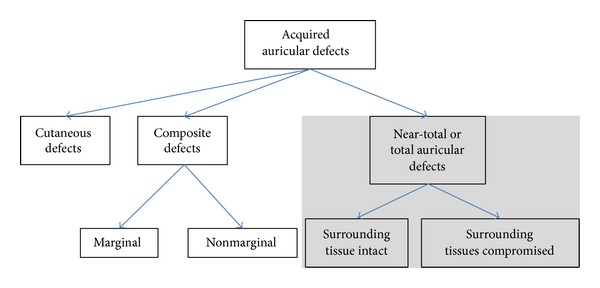
Louis' classification of acquired defects of the ear [5].

Various classifications dealing with acquired auricular defects have appeared in the literature [3, 4]. Since the group of partial defects is highly inhomogeneous, such classifications concentrate on either the localisation of the defect or the tissue affected. Louis et al. [5], for example, generally separate cases into cutaneous defects involving only the auricular skin, composite defects with defects of both the skin and the cartilage, and total or near-total defects requiring the reconstruction of the complete auricle (focus of this article). Composite defects can further be divided into marginal and nonmarginal defects; near-total and total auricular defects can be subdivided into those with healthy/intact surrounding tissue versus damaged surrounding tissue (Figure 3).

## 5. Congenital Deformities

Epidemiologically, Weerda [3] describes one severe deformity for 10,000–20,000 neonates. Although no specific chromosomal abnormality for microtia has been cited, a multifactorial inheritance has been suggested, especially for syndromes such as Franceschetti syndrome or Goldenhar syndrome. However, exogenous factors are also presumed to be causative of malformations in about 10% of cases (thalidomide, rubella embryopathy, other viral infections, alcoholism).

### 5.1. Embryology and Classification

Auricular malformations are based on congenital malformations of the first and second branchial arches surrounding the first branchial cleft. Since a great variety of malformations are possible, a large number of classification systems of congenital defects exist and depend on the author's preference.

In the past, the various classifications found in the literature were mostly based on the degree of deformity and certain anatomical landmarks, for example, Marx (1926) [6] and Tanzer (1977) [7]. Since no international classification system exists, a comparison of studies based on the various terminologies and nomenclatures used is sometimes difficult.

The classification of Weerda [3] combines the suggestions of various authors and provides an overview based on increasing levels of deformity and the necessary surgical intervention (Table 1). In Grade I malformations, most structures of the normal auricle are present. Examples are prominent ears, macrotia, cryptotia, cleft ear, moderate cup ear deformities, earlobe deformities, and other minor auricular deformities [3]. Dysplasia Grade II includes severe cup ear deformities type III and the miniear (concha type microtia) [8]. Some of the ear structures are extant but, for complete reconstruction, additional skin and/or cartilage are needed. In dysplasia Grade III, none of the normal structures are present. This group includes uni- or bilateral rudimentary auricle and anotia [9–11]. In particular, Grade III dysplasia is often associated with changes in the external auditory canal including aural atresia, malformations of the middle ear, and sometimes even dysplasia of the petrous bone with facial anomalies and the facial nerve being affected on the ipsilateral side. In such cases, additional skin and cartilage or other materials are required for total reconstruction.

## 6. Evaluation and Treatment Options

In order to determine the best surgical treatment, a clinical evaluation has to include diverse variables such as patient-related factors (e.g., medical health, medications, and smoking), their reconstructive goals, the pathology of the residual ear (e.g., cause of the defect, size and location of the defect, and type of tissue involved), and the condition of the surrounding hard and soft tissue (e.g., scars, previous infections, and previous attempts of reconstruction). The range of options in auricular reconstruction is wide and includes small interventions, such as primary wound closure, local reconstruction via flap coverage, transplantation of a composite graft from the contralateral ear, and split-skin grafts or full-thickness grafts, up to large interventions, such as full reconstruction including autologous cartilage, alloplastic material, or a prosthesis. As those patients with congenital aural atresia and microtia also suffer from hearing impairment, an optimal hearing function is as important as the aesthetic reconstruction and should therefore be taken into consideration in preoperative planning [12].

### 6.1. Acquired Total Defects

As mentioned above, the reasons for acquired defects are manifold. In the case of tumours, the first goal should always be complete resection with histological margin control; this sometimes makes it difficult to find the best reconstruction technique afterwards. As the highest rates of metastasis from cutaneous spinal cell carcinoma are the lip (14%) and ear (10%), lymphatic drainage, especially preauricular, infraauricular, and post-auricular, always needs to be checked manually and via ultrasound. In cases in which the defect is larger, we prefer the resection of the tumour and the coverage of the defect temporarily with an allogeneous graft. After receiving the final histological result with free margins, we perform the reconstruction. Since partial defects are not the main interest of this paper, an exhaustive summary of all techniques available for the reconstruction of partial defects is beyond the scope of this text. In 2004, Reddy and Zide [13], for example, described various techniques for local reconstruction depending on size and localisation. We refer interested readers to this review [13].

In the case of acquired total defects, the perfect anatomy of the auricle might be difficult to achieve. If enough surrounding soft tissue is available, reconstruction should be the treatment of choice. Therefore, the reproduction of the major landmarks and the correct position, size, and orientation of the auricle are important.

### 6.2. Management of Congenital Deformities (Severe Grade II and Grade III)

#### 6.2.1. Grade II Malformation

In surgery of moderate auricular deformities (Grade II Microtia), also called cup-ear deformity Typ III [10] or concha-type microtia/miniear [14], additional skin and rib cartilage or alloplastic material are necessary for the reconstruction. The ear still displays some structures of a normal auricle but, in severe cup-ear deformity, the auricle is much too small and shows definite hooding of its upper portion. It is also common to find associated dystopia (inferior position and anterior displacement). The auditory canal and middle ear are mostly normal [15].

The creation of a template from the opposite ear, the harvesting of the rib cartilage, and the fabrication of a cartilage framework similar in shape and size to the opposite side are described below in the section on microtia Grade III. In some cases of Grade II malformations, the neoauricle can be sutured to the remaining native auricular cartilage, especially in the lower part of the auricle, if applicable.

#### 6.2.2. Grade III Malformation

The reconstruction of Grade III microtia is a challenging field for facial plastic surgeons and requires total auricular reconstruction. The decision of whether to carry out the reconstruction by using autologous rib cartilage or porous polyethylene frameworks mostly depends on the surgeon and his expertise. With regard to the two materials, only a few special situations are suitable for the use of only autologous material (previous operations with a failed temporoparietal fascial flap) or Medpor (deformities of the thorax). The use of prosthetic techniques should also be reserved for special cases.

## 7. Autologous Material

The reconstruction of the auricle using rib cartilage was described as early as 1968 by Converse [16]. In 1971, Tanzer [17] described a six-stage procedure, modified by Brent [18, 19] and Nagata [14] to a two-stage technique, which is the basis of most current techniques. These procedures were for example, optimised by Weerda [20], Firmin [21], Siegert [22], Staudenmaier [23], and others.

The basic principle of current techniques is the harvesting of the costal cartilage as the first step. After the creation of the framework of the auricle during the same procedure, it is positioned underneath the skin on the planum mastoideum. At an interval of 3 to 6 months, the three-dimensional (3D) projection from the mastoid is accomplished by elevating the neoauricle from the mastoid and creating a posterior auricular sulcus. Often, a third stage is necessary for the fine tailoring of the contours of the auricle.

The indication for reconstruction with autologous rib cartilage depends on the nature and severity of the auricular deformity and on the patient. Starting at the age of 8 to 9 years, it is also a good option in children. At this age, enough cartilage can be harvested and the child is compliant enough to complete the procedure [23]. As mentioned before, for the complete reconstruction of the auricle, at least two operational steps are necessary. In our clinic, we perform a technique modified after Nagata [9–11, 23].

**Figure 4:**
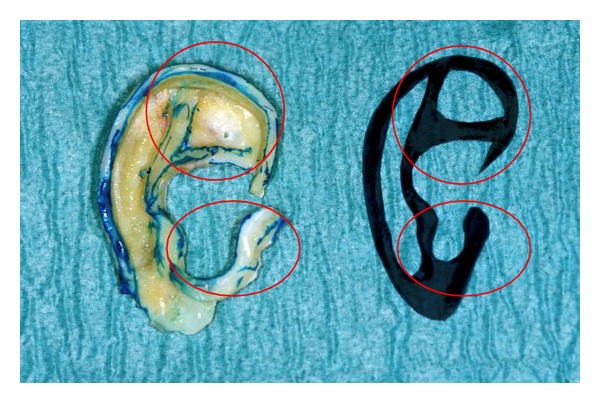
Silicon template and the reconstructed auricle obtained by using the 6th, 7th, and 8th rib, key structures marked in red circles.

Prior to the first operation, a silicone template is created by using the normal auricle on the opposite side to outline the key structures. To accomplish a natural looking auricle, the key structures of the template should always include the complete helix, the antihelix (anterior and posterior crus) with the triangular fossa, the tragus, and the antitragus (Figure 4) [23]. If both ears are affected, a standardised ear can be drawn on a silicone sheet. Various techniques and variable amounts of cartilage are needed depending on the type of the rudimentary auricle or size of the acquired defect.

### 7.1. Procedure

**Figure 5:**
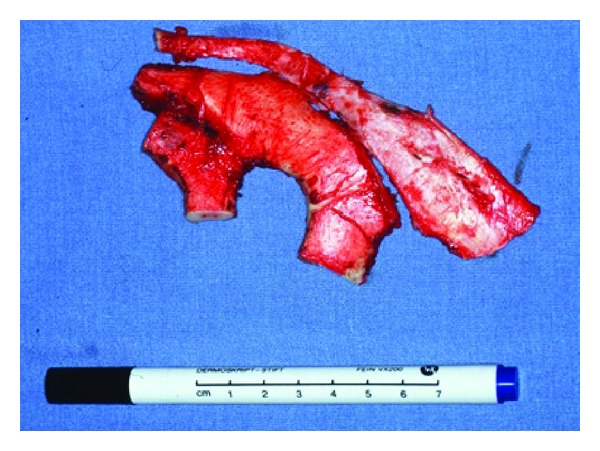
Cartilage from the synchondrosis of the sixth and seventh ribs and also the costal cartilage of the free floating eighth rib.

In the first step, the cartilage from the synchondrosis of the sixth and seventh ribs and the costal cartilage of the free-floating eighth rib are harvested (Figure 5). The basic framework is made from the sixth and seventh ribs giving shape to the triangular fossa and the scapha. To complete the 3D look, we use the floating eighth rib to mould the anterior and posterior crus of the antihelix, the helical rim, the tragus, and antitragus. For the suture of those pieces onto the basic framework, we use a 4/0 transparent nylon suture to prevent translucency of the sutures through the skin (Figure 8 shows the former blue nylon sutures for demonstration purposes).

Simultaneously a subcutaneous pocket needs to be prepared by mobilising and thinning out the skin. According to Nagata [9–11], various lines of incision might be necessary. We prefer the W-shaped line of incision, since it usually is sufficient for the positioning of the framework.

**Figure 6:**
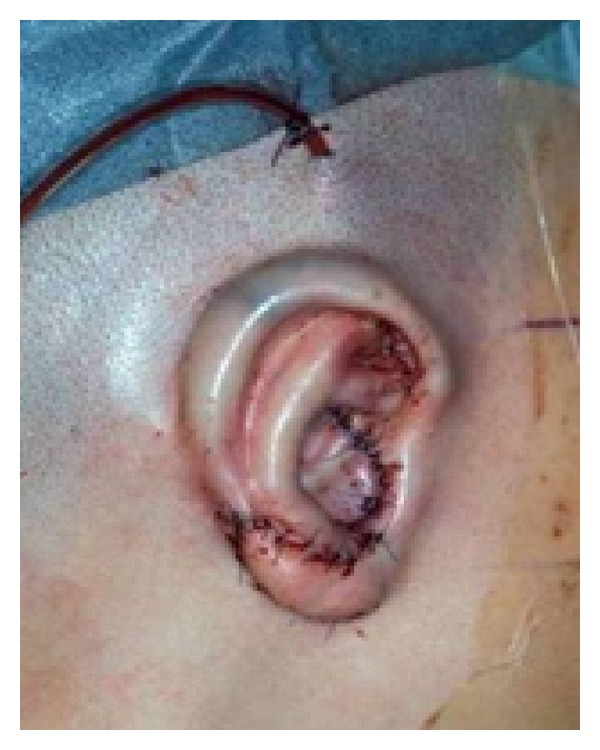
Neoauricle at the end of the first step with a vacuum drain, placed subcutaneously to attach the skin to the cartilage framework.

The initial form of the rudimentary auricle of each patient can widely differ. Moreover, the position and the anatomical variations in the asymmetry of the mandible and the mastoid might play an important role. In some cases, the rudimentary tissue serves to form a neolobule or parts of the auricle, if a smooth junction between the auricular cartilage and costal cartilage can be achieved. Most nonshaping tissues (cartilage or subcutaneous soft tissue) need to be removed in order to create a smooth surface and generate additional skin to cover the neoauricle. After positioning the new cartilage framework underneath the skin on the planum mastoideum, in accordance with that on the opposite side and the surrounding conditions, a vacuum drain is placed subcutaneously to attach the skin to the cartilage framework while suturing the skin. We use a smooth relief dressing instead of mattress sutures additionally to attach the skin to the cartilage and to prevent skin damage by pressure necrosis (Figure 6). This vacuum drain needs to be removed after five days. We place a pain catheter in the intercostal space (donor side) to minimise postoperative discomfort and to minimise the use of oral or intravenous painkillers, especially in children.

**Figure 7:**
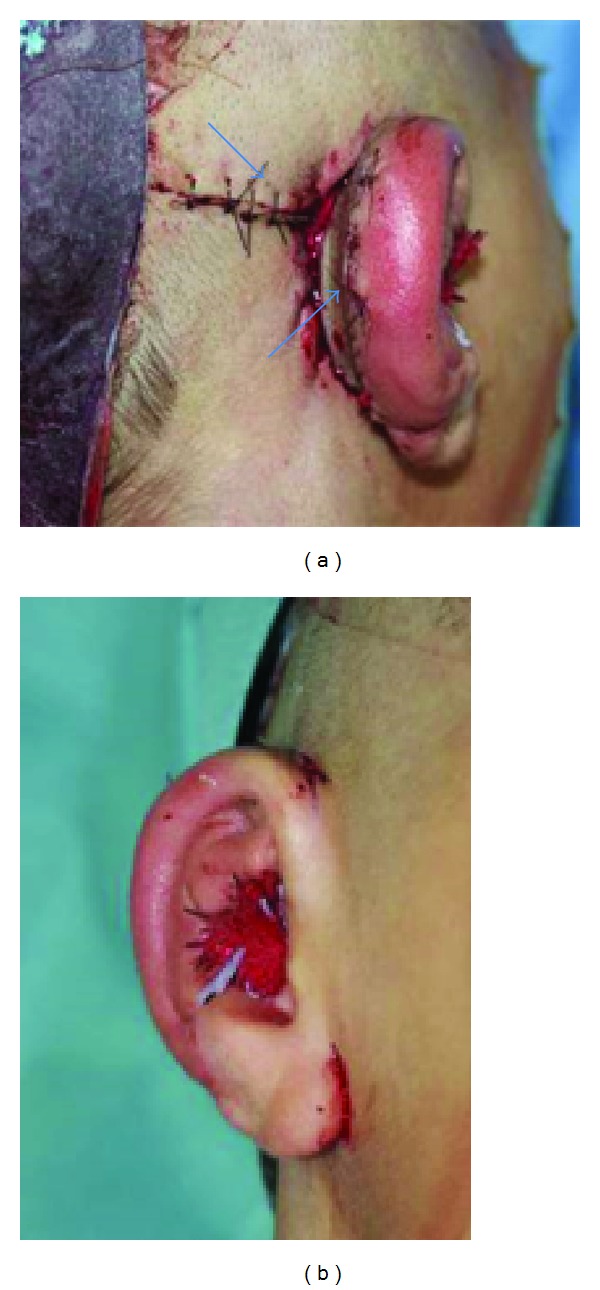
Second step for the three-dimensional projection of the auricle, creation of a retroauricular sulcus using a cartilage wedge. To close the wound a V/Y plasty in the retroauricular fold is used and additional full-thickness skin graft. (*⟶*).

After a healing period of 6–8 weeks, the second stage is performed. This second step is crucial for the 3D projection of the auricle. A retroauricular sulcus is created to elevate the newly formed ear from the side of the head. A piece of cartilage that was subcutaneously positioned in the thoracic bed during the first operation can now be harvested. After incising the skin behind the helix, the bottom part of the neoauricle is mobilised. A wedge of cartilage is positioned underneath and is fixed to the framework by sutures. The cartilage is then encased by an incidentally vascularised dorsal fascia flap (Nagata uses a temporoparietal facial flap). To minimise the defect on the planum mastoideum, we mobilise the surrounding skin (especially in the direction of the neck on the sternocleidomastoid muscle) and fix the skin with a V/Y plasty in the retroauricular fold. The residual crescent-shaped open wound is closed by using a full-thickness skin graft (to minimise the risk of shrinkage) taken from the chest (Figure 7). We also use relief dressing for 7 to 10 days to optimise wound healing.

**Figure 8:**
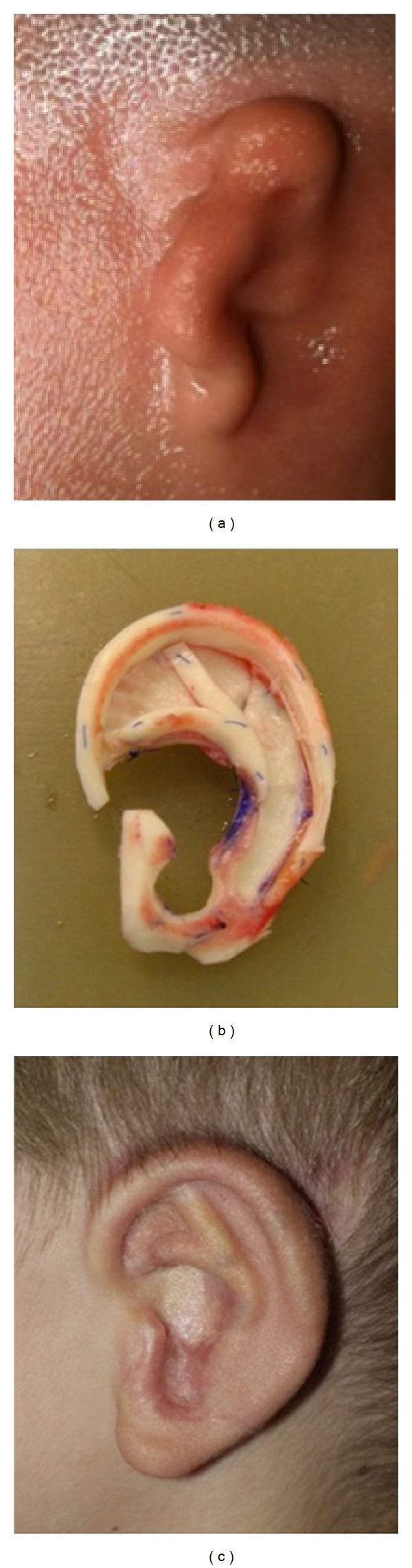
Grade III malformation on the right hand side in a 7-year-old boy, the cartilage framework and the reconstructed auricle after 6 month.

In some cases, an additional operation needs to be performed for the tailoring of the contour of the auricle. This can be performed either under local anaesthesia or under general anaesthesia (Figure 8).

### 7.2. Special Circumstances

Special consideration needs to be taken in patients with difficult periauricular soft tissue conditions. In cases of previous reconstructive attempts, trauma, tumour resection, and especially burns, the local periauricular soft tissue needs to be checked for the movability of the skin, scarring, and perfusion. Often, soft tissue options have been exhausted in previous operations. In these situations, the coverage of the neoauricle with local tissue might be insufficient.

A temporoparietal fascial flap with a free split-thickness skin graft can be useful as coverage in cases of lacking local tissue or vascularisation. This technique requires only one operation, since the retroauricular sulcus is moulded simultaneously. However, the disadvantage of this technique is the slightly different texture and colour of the skin. Alternatively, some authors describe the use of tissue expanders for soft tissue coverage. They use one or two kidney-shaped tissue expanders, place them on the mastoid underneath the subcutaneous fat, and inflate them alternately. After 3 to 6 months of expansion, the construct is positioned in a second operation [24]. In cases of severe damage of the skin and soft tissue and of severe burn damage with destruction of the arteria temporalis superficialis, a prosthetic reconstruction might be the only reasonable solution. When considering the advantages and disadvantages of using autologous rib cartilage, the main issue is the harvesting of the rib cartilage and the comorbidity at the donor site [25]. In a previous study, we reviewed 321 patients needing rib cartilage in cases of auricle or nasal reconstruction. By leaving the inner perichondrium in situ, the risk of a pleural leakage could be minimised. An intercostal pain catheter could minimise postoperative discomfort. In 48 cases, we saw contour irregularity, especially in thin patients, without any functional deficit. Siegert and Magritz [26] have described a resorbable vicryl mesh filled with the rest of the cartilage; this was placed back into the donor site to equalise the contour defect.

The major advantage of this technique is the autologous material used for the reconstruction. It minimises the risk of infection or extrusion of the implant. For many patients, the knowledge of having autologous material implanted is important.

Additionally in normal conditions no temporoparietal fascial flap is needed.

## 8. Alloplastic Material

Since we do not practice this technique in our clinic, the following is based on the recent literature. As an alternative, some surgeons favour using a combination of alloplastic materials, such as porous polyethylene frameworks, and the temporoparietal fascial flap [27–31]. Initially, silicone frameworks were used [32]. Because of high rates of infection, skin perforations, and dislocations, in addition to foreign body reactions and capsule fibrosis, its acceptance has decreased [33]. Thus, porous polyethylene frameworks (Medpor, Stryker, Kalamazoo, MI, USA) have become the most favourable alloplastic frameworks for the majority of surgeons. It is described as a biocompatible thermoplastic. Its open porous structure (pore size between 40 and 200 *μ*m) allows tissue to grow into it.

### 8.1. Operation Technique

The polyethylene skeleton comes in two parts (helical rim and ear base) with the advantage of a greater range in sizing and positioning of the helical rim onto the base portion. Both parts are bound together by heating the plastic or suturing them together. Fine corrections can be made by using a scalpel. Thus, adaptations to the rudimentary ear can be made if required. Before implantation, the construct is soaked in an antibiotic solution to prevent early infection [34]. Reinisch and Lewin [35] have described a method to prevent the exposure of the framework by using a well-vascularised two-layered flap (subgaleal fascia and temporoparietal fascia). Most surgeons practicing this technique with alloplastic material use a temporoparietal facial flap to encase the implant, which is fixed in the required position by stitches. To cover the temporoparietal fascial flap, local skin, and a full-thickness skin graft from the contralateral retroauricular region, the abdominal wall or groin region is used. As in autologous constructs, vacuum drains are positioned underneath the flap to suck away wound secretion and to ensure a close contact between the skin, fascia flap, and skeleton. This technique only requires one operation. Complications described in the literature for alloplastic material include the risk of infection and extrusion [29].

In addition, special risks of using the temporoparietal fascial flap concern the preservation of the superficial temporal artery and vein to ensure an axial blood supply in order to obtain a reliable flap; alopecia is another risk [36].

## 9. Prosthetic Restoration

The third possibility for rehabilitation of facial aesthetics is the prosthetic reconstruction technique [5, 37–39]. Especially in cases of traumatic loss of the complete auricle with large defects and deep soft tissue injuries, but also after malignant tumour resection in older patients, prosthetic restoration is a good alternative to autogenous reconstruction. Even in cases of failed autogenous reconstruction, osseointegrated alloplastic ear reconstruction can be performed as second-line therapy. Since the aesthetic results become more favourable, the prosthetic restoration is a good option for first-line therapy as well, for example, for patients you withhold or are not able to undergo extensive multistep approach necessary for total auricular reconstruction surgery.

The indications for osseointegrated alloplastic ear reconstruction are [37]

- major cancer resection;
- radiotherapy;
- severely compromised tissue;
- patient preference;
- failed autogenous reconstruction;
- potential craniofacial anomaly;
- poor operative risk.

**Figure 9:**
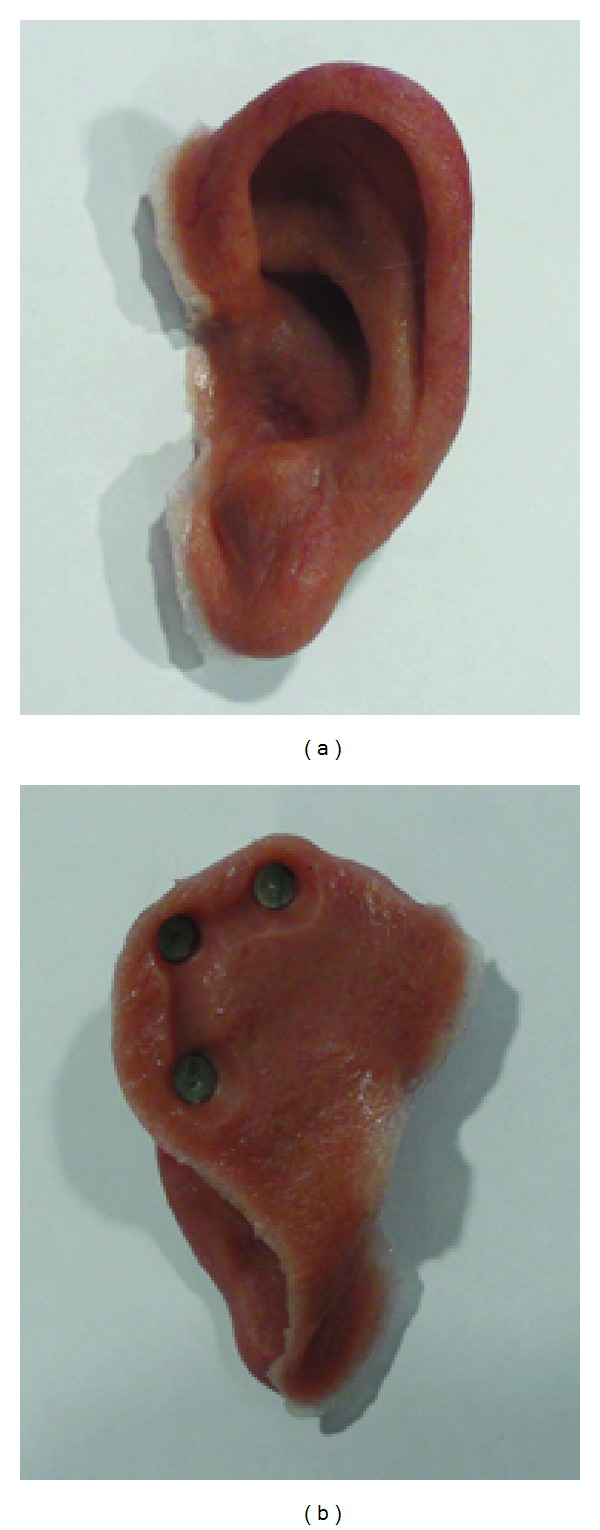
Silicone auricular prosthesis with integrated magnets. Note the very thin anterior margin camouflaging the border to the patient's own skin.

**Figure 10:**
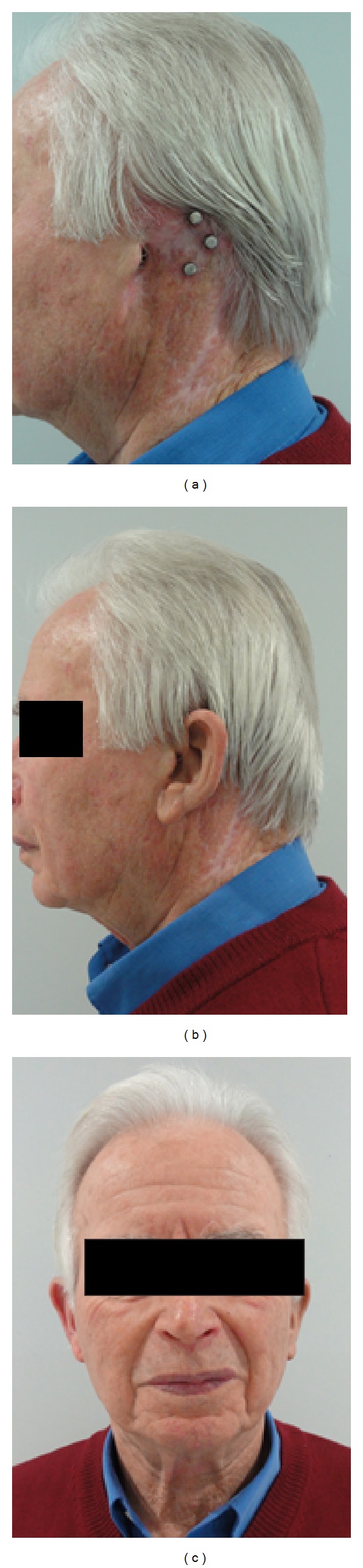
Auricular reconstruction via prosthesis and osseointegrated implants after complete auricular resection due to skin cancer. The prosthesis is held in place by use of integrated magnets at both the implant screws and the silicone auricle.

The alloplastic ear is made of silicone and can be formed mirror inverted to the contralateral intact ear. In case of bilateral aural atresia, the prosthesis can be made referring to a parent's ear and with respect to the proportion of the patient's skull. Intrinsic silicone colour matching is performed based on the patient's unique skin tone (Figures 9 and 10). Various means of attaching the prosthesis to the skin are available. The prosthesis is hold by the original defect hooked into a bony edge, the prosthesis is stuck on the defect by an adhesive, or the prosthesis is attached to surgically implanted and osseointegrated titanium screws. The first option is a good choice for defects of the nose, while, in cases of atresia or total loss of the auricle, a mostly flat surface does not offer any overhanging edges. The latter both techniques are commonly used for ear prosthesis. With the use of adhesives, no surgery is needed at all. Although there are a few disadvantages with the use of implants, which include the possibility of infection and inflammation around the screws, the advantages outweigh the disadvantages. With the use of implants, it is much easier for the patient to place the prosthesis properly even without the help of a mirror. Due to less mechanical load of the prosthesis, the life span of the device is improved. The biggest advantage is the improved retention of the prosthesis to the head, especially in case of shear forces. Particularly when the patient is doing sport, adhesives tend to come off with sweating.

The surgical procedure is usually performed in two stages. The first step includes excision of any auricle remnant or excision of an unfavorable result of failed previous surgical auricular reconstruction and placing the implant screws (e.g., Ponto implant, Oticon Medical AB, Askim, Sweden) to the bone. Then the implants are loaded with special cover screws and the wound is closed. After wound, healing the second step follows a few weeks later. During step two, the implants are uncovered with use of a skin punch and skin penetrating magnetic inserts (e.g., Titanmagnetics, steco-system-technic, Hamburg, Germany) are connected to the implants. While step two can easily be done in an outpatient setting with local anaesthesia, the first step should be performed under general anaesthesia.

**Figure 11:**
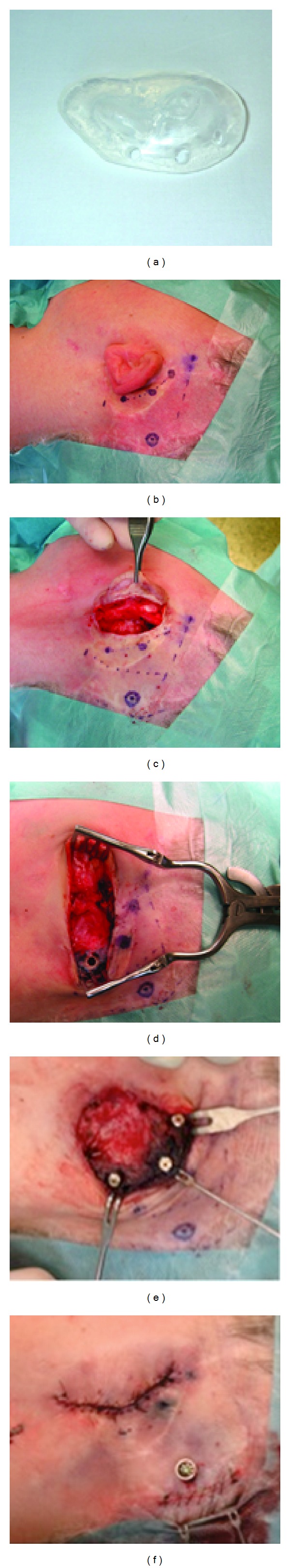
(a) Sterile silicone surgical guide. (b) Failed previous autogenous reconstruction with marks for both prosthetic and bone anchored hearing device implants. (c) Excision of auricular remnants. The incision is all at once used for placing the prosthetic implants. (d) After preparing a periosteal flap the implant holes are drilled. (e) The implants are loaded with cover screws until surgical step two. (f) Closed wound with abutment for bone anchored hearing device in place.

**Figure 12:**
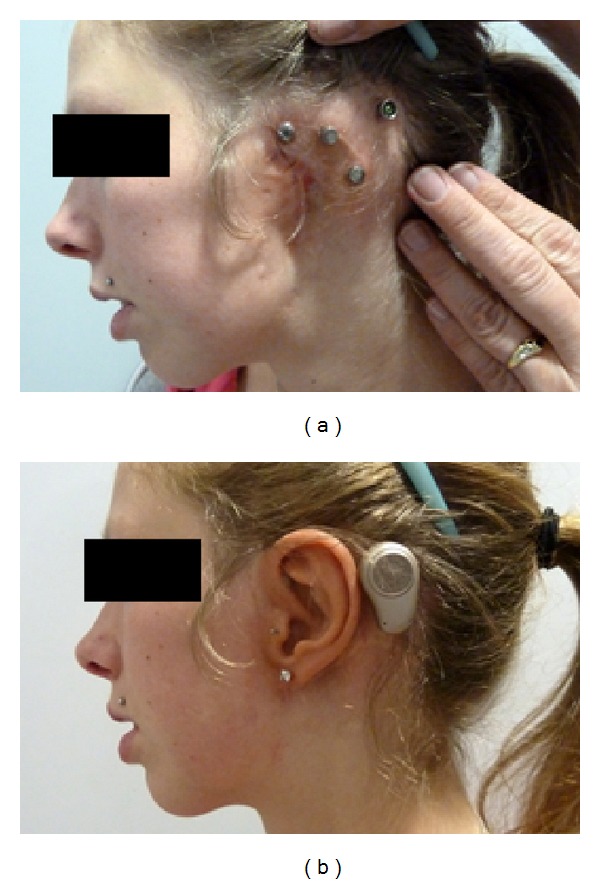
Same patient as in Figure 11 with and without implant-retained prosthesis in place. Note the additional bone anchored hearing device (Ponto Pro, Oticon Medical AB, Askim, Sweden) on the right side.

If any excision of auricular remnants is needed, this incision can mostly be used for placing the implants (Figure 11(c)). Otherwise, an extra incision creating a skin flap has to be made. An individual preoperative surgical guide is used to find the proper implant position (Figure 11(a)). In case of congenital microtia with atresia of the ear canal hearing rehabilitation can be achieved by placing an additional implant for a bone conducting hearing device (Figure 11(f)). In all cases a preoperative CT scan of the temporal bone and the middle ear anatomy is favourable.  Figure 12 shows the same patient as in Figure 11 with and without the implant-retained prosthesis in place. In addition the patient wears a bone anchored hearing device (Ponto pro, Oticon Medical AB, Askim, Sweden).

## 10. Functional Hearing Reconstruction

**Figure 13:**
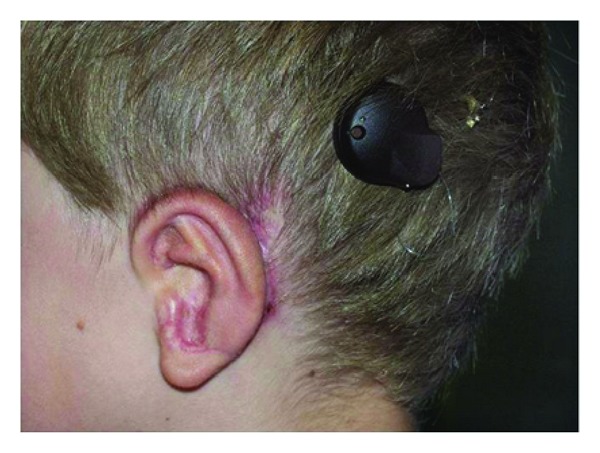
Young patient with Grade III microtia and aural atresia after auricular reconstruction via rib cartilage and implantation of a vibrant soundbridge hearing device.

As mentioned above, an optimal hearing function is essential for a patient's quality of life [12]. As patients with congenital aural atresia and microtia particularly suffer from hearing impairment, optimal hearing function is as important as aesthetic reconstruction and needs to be taken into consideration during preoperative planning. The reconstruction of the auditory canal in severe Grades II and III malformations with aural atresia for aesthetic and also functional reasons remains a challenging subject [40]. Within the last few years, other approaches such as bone-anchored hearing devices or active middle ear implants (MEI) have been described for functional repair. In a review based on 107 publications, Nadaraja et al. [41] have recently reported that hearing outcomes after the use of osseointegrated bone conduction devices are superior to atresiaplasty results. In a study by Kiefer and Staudenmaier [42], fifteen patients underwent implantation of an active MEI (vibrant soundbridge). The vibrating element, the floating mass transducer (FMT), was coupled either to the round window, stapes, or oval window or incus according to the individual middle ear situation. In 14/15 patients, a satisfactory functional result could be achieved (<30 dB pure-tone audiometry) making the soundbridge a valuable option for functional reconstruction. In combination with the reconstruction of the auricle via autologous cartilage, we recommend such implantation during the second operation step (Figure 13).

## 11. Tissue Engineering of Cartilage

Tissue engineering is a promising field for the repair or replacement of all kinds of damaged tissue. Green and Dickens [43] described the first culture of cartilage in 1972. Since then, diverse groups from all over the world have concentrated on the tissue engineering of cartilage. Nevertheless, the clinical outcome still falls behind the promising expectations concerning cell vitality, homogeneous cell seeding in 3D scaffolds, form stability and eventual vascularisation of the cultured constructs for the supply of nutrients and oxygen [44].

To culture ear cartilage, pluripotent stem cells or isolated primary chondrocytes can be used. The cell population then has to be expanded in vitro and seeded onto suitable scaffolds to engineer a functional auricle. This approach might avoid donor site morbidity and donor site morbidity associated with harvesting costal cartilage and hand carving an auricle [45]. Work with stem cells is possible by using a broad range of tissue as the cell source, although the differentiation of the cells is often incomplete. Remnants of the auricle or the contralateral auricle could ideally be used as donor tissue for autologous chondrocytes. A small biopsy of the nasal septum is also possible. However, we should bear in mind that nasal cartilage is less elastic and tends to calcify in comparison with elastic cartilage. The use of extremely small pieces of cartilage necessitates many cell passages in order to achieve sufficient cell numbers. This leads to dedifferentiation of the chondrocytes. A 3D shaping of the cultured cartilage is possible by using synthetic (e.g., poly-e-caprolactone, polyglycolic acid, poly-L-lactide acid, or polylactic-co-glycolic acid) or biological (e.g., collagene, alginate or fibrin gel, or chitosan) scaffolds. It is also possible to combine a naturally derived scaffold with a synthetic material (e.g., a flexible wire) to prevent construct shrinkage [46].

**Figure 14:**
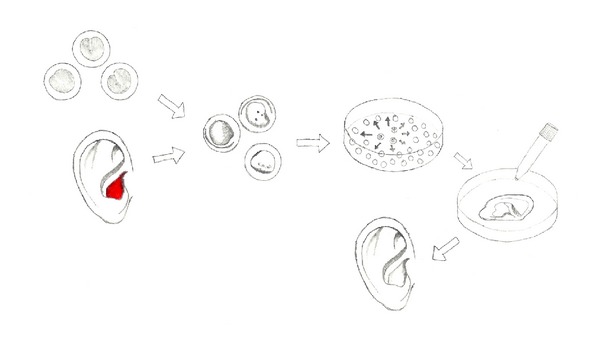
Auricular tissue engineering. Pluripotent stem cells are chondrally differentiated or alternatively autologous chondrocytes are isolated from an auricular biopsy (e.g., from the concha (red) or auricular remnants). After expansion, they are seeded onto an auricular-shaped scaffold and cultivated in 3D culture. Afterwards, the cultured auricle can be implanted. For better results, neovascularisation is recommended [44]. Figure modified after Bichara et al. [45].

A tissue-engineered auricle could be custom designed to match the contralateral auricle and the appearance of the patient by using rapid prototyping [47].  Figure 14 shows potential methods for auricular tissue engineering.

**Figure 15:**
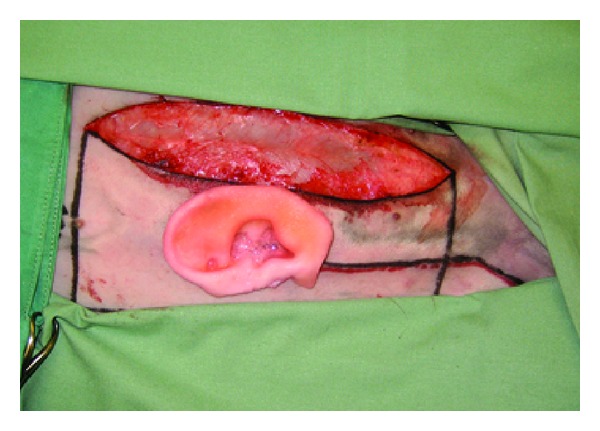
Tissue engineering of chondrocytes with the prefabrication of a 3D cartilage auricle construct (polycaprolactone-based polyurethane scaffold) before implantation in the rabbit.

Our previous and recent experimental studies have led to good results in the tissue engineering of cartilage with the prefabrication of 3D cartilage constructs (polycaprolactone-based polyurethane scaffolds) (Figure 15) leading towards a tissue-engineered auricle [44, 48]. In an autologous rabbit model, cartilage cell constructs were neovascularised by means of vascular loops implanted microsurgically and placed subcutaneously into a skin flap. After a minimum of 21 days, the 3D construct could be freely transplanted by microsurgery. This technique would allow cases of severe damage to the skin and/or soft tissue to be repaired by reconstruction. After explantation, the constructs were shown to have formed engineered cartilaginous tissue with cartilage-specific extracellular matrix components (GAG, collagen type II), even in the centre, and the constructs presented good neovascularisation [44]. The procedure seems to be a highly promising alternative for clinical practice but further investigations are warranted.

## 12. Discussion

Auricular reconstruction is one of the greatest challenges in facial plastic surgery and, with the advances in both surgical techniques and biotechnology, a variety of options can be taken into consideration by surgeons and patients. Initially described by authors such as Converse [16], Tanzer [17], and Brent [18] dealing with large numbers of patients, auricular reconstruction was later developed by Nagata [9–11] and Firmin [21], also with large numbers of patients, and has now been reduced to an operation of two stages.

Reviewing the literature, there have also been many other authors reporting on auricular reconstruction within the last three decades with big advances in the autologous, alloplastic but also prosthetic reconstruction. Most surgeons still favour autologous reconstruction; many have adopted the Nagata approach in many variations. These variations include the shaping of the framework, flaps to cover the framework, and the amount of cartilage that is used. For the 3D projection of the ear, Nagata uses a wedge of cartilage that is positioned on the underside to elevate the framework and is fixed to the framework by sutures. He employs a temporoparietal fascial flap to cover the cartilage [49]. We utilize a vascularised flap to cover the cartilage. The flap is thinner and we can thus save the temporoparietal fascial flap in cases of complications such a skin necrosis.

In 1966, Cronin [32] introduced an alloplastic material (silastic framework). He described complications such as extrusion and infection of the framework. More recently, porous polyethylene frameworks have been employed for auricular reconstruction at various medical centres [39, 42]. The alloplastic framework should be covered by a well-vascularised flap to prevent extrusion. The choice of whether to use a temporoparietal fascial flap (risk of alopecia and scarring) depends on the surgeon. Two great advantages propagated by the advocates of this method are the one-stage procedure and the lack of donor site morbidity (thoracic bed). On the other hand, the donor side morbidity by harvesting large pieces of skin should not be overlooked. Moreover, the harvesting of the temporoparietal fascial flap with the risk of alopecia is an important aspect to be borne in mind.

The third possibility is reconstruction via a prosthesis. The recent techniques with osseointegrated titanium screws offer advantages such as the improved attachment of the prosthesis, ease of use, and proper positioning of the prosthesis [5]. Nevertheless, the use of a prosthesis is still preserved for special situations such as failed autogenous/alloplastic reconstruction with severe soft tissue defects or scarring or in older patients after an acquired total auricular defect (especially for patients wearing spectacles).

Recent authors focus on the outcome, the benefit to the patients quality of life and their psychological improvement after the operation (either with rib cartilage or Medpor), retrospectively [30, 50] and prospectively [51], a topic that seems to have become an important issue within the last few years. As Sivayoham and Woolford [52] mentioned in their article, “it is becoming increasingly important that we can justify the commissioning of operations such as total ear reconstruction, not just with the intuitive observation that of course it is desirable to have two ears, but with high-quality evidence.” Braun et al. [30] and Soukup et al. [50] have used the Glasgow inventory scale, which is a health-related quality-of-life assessment tool, to detect patient satisfaction after auricular reconstruction (autologous or alloplastic material). Both studies show a positive health-related quality-of-life benefit (total score and social health subscale) in the total reconstruction either with autologous material (Soukup) or Medpor (Braun). Steffen et al. [51] have shown comparable results by using a different questionnaire.

Younis et al. [53] report on patient satisfaction (*n* = 20) after reconstruction via a Branemark-type bone-anchored ear prosthesis, after 14/20 patients had undergone a failed auricular autologous reconstruction; the results reveal an overall acceptance of the aesthetic appearance but also multiple chronic skin problems (15/20 patients) and other implant problems.

**Figure 16:**
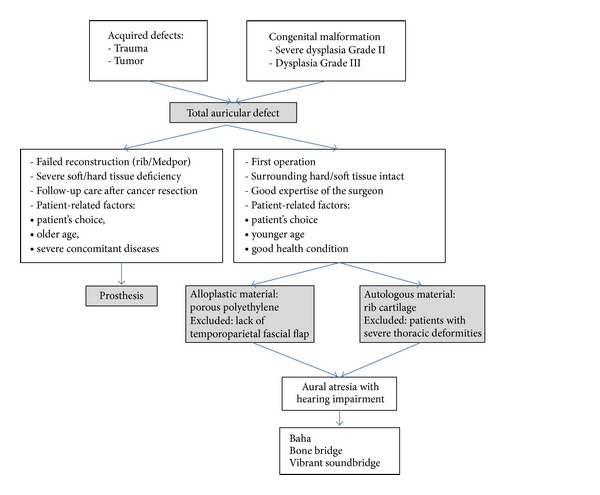
Guidelines for total auricular reconstruction.

These findings once more display the use of autologous or alloplastic material as the method of choice, with reconstruction via a prosthesis only being employed in special situations.  Figure 16 provides a guideline to the decision-making process in the treatment of total auricular defect.

## 13. Conclusion

The field of auricular reconstruction remains a great challenge to facial reconstructive surgeons. The method of choice for such reconstruction depends not only on the patient's pathology and the state of the local tissue and skin, but also on the preferences of the facial plastic surgeon and the patient. Various possibilities of reconstruction exist, such as autologous rib cartilage, alloplastic material, or prosthetic restoration. In smaller defects, local soft tissue and skin might suffice to cover the defect by various flap techniques. The recent literature indicates that current practice still favours the use of costal cartilage grafts, although many advocates of alloplastic implants can be found. The choice often depends on the preference of the author and whether the pros or cons of each technique are highlighted. In our clinic, we prefer reconstruction via rib cartilage and have achieved good results with this method over many years. However, as mentioned above, the use of a prosthesis might be the best treatment in special situations. Irrespective of the technique that is used, the implantation of a bone-anchored hearing device or an active middle ear implant should be taken into consideration preoperatively in order to restore complete hearing in cases of congenital atresia.

## Figures and Tables

**Table 1:** Weerda's classification of auricular malformations based on an increasing severity of the malformation (shortened) [3].

Degree of dysplasia	Definition	Subgroup
I: Low-grade malformations	General: most of the structure of a normal auricle are present Surgical: additional skin and cartilage are only occasionally required for reconstruction	(i) Prominent auricle (ii) Macrotia (iii) Cryptotia (pocket ear) (iv) Cleft ear (transverse cleft) (v) Scaphoid ear (vi) Stahl's ear (vii) Satyr ear (viii) Small deformities (ix) Lobule deformities (x) Tanzer's types I, IIA, and IIB cup-ear deformities

II: Grade II microtia; moderate malformations	General: the auricle still displays some structure of a normal auricle Surgical: additional skin and cartilage required for partial reconstruction	(i) Tanzers's type III cup-ear deformity (ii) Miniear (Hypoplasia of the upper, middle of lower auricle)

III: Grade III microtia with anotia; severe malformations	General: structures of a normal auricle no longer present Surgical: additional skin and cartilage required for total reconstruction	(i) Unilateral Grade III microtia (Nagata's lobule type microtia) (ii) Bilateral Grade III microtia (iii) Anotia (iv) Normally congenital aural atresia will be found
